# Do Leaf Cutting Ants Cut Undetected? Testing the Effect of Ant-Induced Plant Defences on Foraging Decisions in *Atta colombica*

**DOI:** 10.1371/journal.pone.0022340

**Published:** 2011-07-20

**Authors:** Christian Kost, Martin Tremmel, Rainer Wirth

**Affiliations:** 1 Department of Bioorganic Chemistry, Max Planck Institute for Chemical Ecology, Jena, Germany; 2 Department for Plant Ecology and Systematics, University of Kaiserslautern, Kaiserslautern, Germany; University of California Davis, United States of America

## Abstract

Leaf-cutting ants (LCAs) are polyphagous, yet highly selective herbivores. The factors that govern their selection of food plants, however, remain poorly understood. We hypothesized that the induction of anti-herbivore defences by attacked food plants, which are toxic to either ants or their mutualistic fungus, should significantly affect the ants' foraging behaviour. To test this “*induced defence hypothesis*,” we used lima bean (*Phaseolus lunatus*), a plant that emits many volatile organic compounds (VOCs) upon herbivore attack with known anti-fungal or ant-repellent effects. Our results provide three important insights into the foraging ecology of LCAs. First, leaf-cutting by *Atta* ants can induce plant defences: Lima bean plants that were repeatedly exposed to foraging workers of *Atta colombica* over a period of three days emitted significantly more VOCs than undamaged control plants. Second, the level to which a plant has induced its anti-herbivore defences can affect the LCAs' foraging behaviour: In dual choice bioassays, foragers discriminated control plants from plants that have been damaged mechanically or by LCAs 24 h ago. In contrast, strong induction levels of plants after treatment with the plant hormone jasmonic acid or three days of LCA feeding strongly repelled LCA foragers relative to undamaged control plants. Third, the LCA-specific mode of damaging leaves allows them to remove larger quantities of leaf material before being recognized by the plant: While leaf loss of approximately 15% due to a chewing herbivore (coccinelid beetle) was sufficient to significantly increase VOC emission levels after 24 h, the removal of even 20% of a plant's leaf area within 20 min by LCAs did not affect its VOC emission rate after 24 h. Taken together, our results support the “*induced defence hypothesis*” and provide first empirical evidence that the foraging behaviour of LCAs is affected by the induction of plant defence responses.

## Introduction

Leaf-cutting ants (LCAs) are among the most polyphagous and voracious herbivorous insects known of the Neotropics, cutting up to 15% of the standing leaf crop [1], [2] and up to 50% of the species available in the vicinity of their colonies [2], [3]. The ants use the harvested leaf material to cultivate a symbiotic fungus that in turn produces protein-rich food bodies - the sole food for the ants' larvae. The sophisticated habit of cultivating a symbiotic fungus is generally believed to be key to the LCAs' tremendous ecological success [2]. Although LCAs attack an enormous diversity of plant species, their choice of food plants is highly selective. While a number of leaf characteristics such as nutrient content, leaf toughness and the amount of compounds toxic to the ants or the fungus have been identified to affect their choice of plants [4]-[9], several fundamental issues regarding the foraging ecology of these ants remain obscure.

LCA colonies are largely sessile and explore their home range via a complex system of foraging trails [10]. As central-place foragers, LCA are expected to minimize the costs of their leaf harvest (i.e. time spend during foraging, trail construction and maintenance) and at the same time maximise their gain in terms of energy intake. This idea, which is encapsulated in the so-called ‘*optimal foraging theory*’ (OFT, [11]), predicts for an environment with a patchy distribution of food resources that once a suitable food plant has been detected by scouting ants, foraging workers will defoliate it to a point, at which the rate of food intake drops below the average rate for the rest of the habitat. This prediction has been termed ‘*marginal value theorem*’ [12]. In doing so, the ants would maximise the growth of their symbiotic fungus, and hence of the whole ant colony and - at the same time - reduce energetic costs incurred by the search for new food plants such as the establishment of new foraging trails (e.g. [13]). Another prediction made by the OFT is that given two plant individuals with equal leaf qualities (e.g. same plant species), foraging ants should always select the plant individual that is closest to the nest to reduce travelling time.

In contrast to these predictions, two observations have puzzled researchers for decades: First, it has frequently been observed that foraging LCA colonies stop exploiting a tree long before it has been completely defoliated, although it might still be a profitable food resource [14]. Such attacks rarely last longer than one day and only few plant individuals face persistent defoliation for more than one week [2], [10], [15]. Second, LCAs often travel greater distances to harvest leaves from trees, even though conspecific plants with presumably equal leaf qualities are available much closer to the nest [16], [17].

Several hypotheses have been put forward to explain these phenomena: First, the ‘*resource conservation hypothesis*’ [16] predicts that LCA-colonies ‘conserve’ preferred resources by limiting the inflicted damage. Second, the ‘*nutrient balance hypothesis*’ [18] argues that LCAs should aim at selecting a variety of leaves from different plants to provide a suitable mix of nutrients to optimize fungal growth. Third, LCA defoliation patterns could be explained by the OFT, given a patchy distribution of resource qualities even among conspecific plants [15], [17]. Finally, and along similar lines as the previous hypothesis, the observed pattern could be explained by the fact that the continuous removal of leaf material from a plant may lead to the induction of anti-herbivore defences [19], [20]. This possibility, hereafter referred to as ‘*induced defence hypothesis*’, predicts that herbivory results in an immediate or delayed activation of plant defences, which may adversely affect the same or future generations of the attacking herbivores. If the cutting of leaves induced plant defences, LCA foraging would generate a dynamic mosaic of plants at varying induction levels in their foraging area that might explain the abovementioned pattern.

The spectrum of mechanisms plants use to protect themselves against herbivores ranges from direct defences that immediately affect the attacking herbivores (e.g. mechanical barriers or toxic compounds [21]) to indirect defences that facilitate ‘top-down’ control of herbivore populations by attracting the herbivore’s natural enemies [22]. One of the most widely distributed defence mechanisms that can act both directly [23], [24] and indirectly [25]–[27] is the emission of herbivore-induced volatile organic compounds (VOCs). Both direct and indirect plant defences can either be expressed constitutively or be induced following herbivore attack. Induction of plant defences is usually regulated by the octadecanoid pathway [28], [29], in which jasmonic acid (JA) acts as the central signalling molecule [30]. External application of JA-solution induces defence mechanisms including the release of VOCs [31], thereby providing a convenient tool that allows defence induction without the need to damage leaf tissues.

To test the ‘*induced defence hypothesis*’, we selected lima bean (*Phaseolus lunatus*) as a model plant that likely is also attacked by LCAs in nature [32], [33] and is readily harvested by our laboratory *Atta* colonies. This plant species is very well-known for increasing its emission rate of VOCs following herbivore attack (for review see [22]) and JA application. Moreover, the blend of VOCs emitted from JA-treated plants closely resembles the one released after herbivore damage both quantitatively and qualitatively [34]. Moreover, several compounds of the volatile blend emitted from induced lima beans are known to be toxic to LCAs and/or their mutualistic fungus, or exhibit a general fungicidal activity and hence act as a direct defence in this context. For example, (*E*)-β-ocimene was highly repellent against *Atta cephalotes* workers [35], β-caryophyllene repelled ants of the same species and inhibited the growth of their fungus [6], [7], and both (*R*)-(-)-linalool and methyl salicylate have general fungicidal properties [36], [37].

Hence, we speculated that if the ‘*induced defence hypothesis*’ was correct, leaf damage by LCA should induce anti-herbivore defences in the attacked plant including the emission of VOCs. These, in turn, might function as a direct defence against both ants and their symbiotic fungus and therefore repel ant workers. If this was true, two requirements should be met: i) LCA damage should induce VOC emission in lima bean plants, and ii) LCA workers should be repelled from induced lima bean plants. We verified these predictions by both measuring the VOCs emitted from LCA-damaged and JA-treated lima beans and in dual choice assays, in which foraging workers of the LCA *Atta colombica* were simultaneously exposed to differentially treated lima bean plants and untreated control plants.

## Materials and Methods

### Study Species

Bioassays were conducted with three laboratory colonies of LCAs (Formicidae: *Atta colombica*). The ant colonies originated from Gamboa, Panama, were about 6 years old and their fungus gardens occupied about 15 l each. Lima bean plants (Fabaceae: *P. lunatus*) L. Ferry Morse cv. Jackson Wonder Bush were raised in a greenhouse with a 12 h photoperiod (daylight and artificial light; 7:00–19:00) and minimal night temperatures at 20°C. Plants were grown in plastic pots with 14 cm in diameter. Plant used for the bioassays were 3–6 weeks old and had developed 2–5 leaves.

### Experiment 1: LCA Damage (24 h)

To analyse whether LCA damage induces significantly increased emission rates of VOCs within 24 h post ant attack and if so, whether this affects the ants' foraging decision, test plants were subjected to one of five different treatments: (I) Exposure of lima bean plants to a singular event of LCA-herbivory until the ants had removed approximately 20% of the leaf area (duration ca. 20 min). (II) Simulation of LCA-herbivory (i.e. removing leaf pieces of similar size as LCA-cut fragments) with nail scissors by removing 20% of the leaf area. This treatment was performed to exclude potential error sources resulting from (a) ants preferring previously visited plants, and (b) VOC induction by potential, yet unknown, elicitors from the ants' oral secretions. (III) Piercing of the whole leaf area with a pincushion to disrupt a larger number of cells without causing wounded leaf margins, that LCAs prefer as starting points for new cuts [4, personal observation]. (IV) Spraying the plants with a 1 mmol aqueous solution of JA until the leaf-surfaces were completely covered [38]. (V) Exposure of plants to LCA herbivory as described above with subsequent JA application similar to treatment IV to test for additivity of the two treatments. Untreated plants served as controls. All treatments were applied simultaneously to one set of plants within 20–30 minutes. After that, plants were placed back into the greenhouse to allow the induction of plant defences. The next day (i.e. after 24 h), the so-treated plants were used to quantify the amount and composition of the VOCs emitted as well as for dual-choice experiments with LCA colonies (see below).

### Experiment 2: Beetle Damage (24 h)

As a control experiment that aimed at quantifying the amount of HI-VOCs emitted from lima bean plants that have been damaged by an herbivore with chewing mouthparts within 24 h, 16 plants were exposed to five Mexican bean beetles each (Coccinellidae: *Epilachna varivestis* Mulsant). This was done by enclosing bean tendrils for 24 h in bags made of nylon nets into which beetles have been inserted. Sixteen control plants were left untreated and six plants treated with JA as described above. After 24 h, beetles were removed, plants were bagged and the emitted VOCs collected and measured as described below.

### Experiment 3: Time Course of LCA Damage (0–4 d)

The question whether a prolonged LCA-defoliation of lima beans results in significantly increased emission rates of VOCs and if so, whether this also affects LCA foraging was addressed by increasing the damage level applied by LCAs to plants step-wise over the course of four days. Plants were left untreated (control) or exposed to LCA foragers until approximately 20% of the leaf area of the plants has been removed (1 d). Other plants were exposed to LCA foragers daily over the course of either 2, 3 or 4 days. On each day, ants were allowed to remove 20% of the available leaf area (2–4 d), which in general lasted approximately 20–30 min. After each LCA treatment, plants were placed back into the greenhouse to allow defence induction. After 24 h, the so-treated plants were either treated again or used to quantify the amount and composition of the VOCs emitted as well as for dual-choice experiments with LCA colonies (see below).

### Measurement of VOC Production

After application of the respective treatments, plants were immediately bagged in a PET foil (‘Bratenschlauch’, Toppits, Minden, Germany) that does not emit detectable amounts of volatiles and placed in the greenhouse for 24 h. During this time, the emitted VOCs were collected continuously on charcoal traps (1.5 mg charcoal, CLSA-Filters, Le Ruissaeu de Montbrun, France) using air circulation as described previously [39]. After 24 h, leaf areas of the plants were estimated with a leaf area analyzer (Experiments 1 and 3; LI 3100, Li-Cor, Nebraska, USA) or the dry weight of the leaves determined (Experiment 2). Volatiles were eluted from the carbon trap with dichloromethane (40 µl) containing 1-bromodecane (200 ng µl^−1^) as an internal standard. Samples were stored at −20°C and analyzed on a GC-Trace mass spectrometer (Thermo Finnigan: www.thermofinnigan.com) according to Koch *et al.* [40]. Individual compounds (peak areas) were quantified with respect to the peak area of the internal standard and related to the leaf area of the measured plant (Experiments 1 and 3) or the dry weight of the emitting leaves (Experiment 2).

### Dual Choice Bioassays

To quantify attractiveness of treated lima bean plants to foraging workers, bioassays were performed in an open plexiglass arena (60×60×10 cm). For each replicate, two potted plants (i.e. untreated control and treated plant) were inserted into the arena by placing them in two holes in the base of the arena that were equally spaced from the centre. The holes were covered with a divided plastic disc sparing the stem of the plant. The ants entered the arena via a tube that ended in the centre of the arena. Harvested leaf area was estimated by outlining the leaf contours of test and control plants before and after the feeding trial. A trial was stopped after the ants had removed approximately 20% of the leaf area of one of the two plants. After each trial, the arena was wiped with 70% ethanol to remove residual ant pheromones that might influence subsequent experiments. This bioassay was replicated at least 11 times per plant treatment and at least two trials per plant treatment were performed for each of the three colonies. Preference was calculated as a ‘*mean acceptability index’* (MAI). For this purpose, the leaf area removed from the focal plant was divided by the area removed from the sum of both test and control plants. The resulting values range from 0 (total rejection of test plant) to 1 (100% preference of the test plant).

### Statistical Analysis

MAI data was analysed with a mixed-effect model with ‘treatment’ as fixed and ‘ant colony’ as random variable. MAI values were rank- or squareroot-transformed to meet the test assumption of homogeneous variances. The effect of treatments on the total amount of VOCs emitted was evaluated with a Welch test due to heterogenous variances. Differences between treatments were analysed with Tamhane's post-hoc test. A Spearman rank correlation was applied to test the relationship between i) the amount of volatiles emitted from a given plant and the corresponding MAI measured, or ii) the amount of volatiles emitted from a given plant and the amount of damage received after feeding of Mexican bean beetles. These analyses were done using SPSS 17.0 (SPSS Inc., Chicago, USA).

The similarity between VOC profiles emitted from differentially treated plants was estimated as the Euclidean distance in a multidimensional space, in which each VOC represented one dimension. For this, a Euclidean dissimilarity matrix was calculated from the dataset, which was then subjected to an agglomerative hierarchical clustering method (unweighted pair-group method with arithmetic mean, UPGMA) that can be represented graphically by means of a dendrogram. This analysis was performed using the R statistical package [41].

## Results

### Volatile Emission 24 h after LCA Damage

**Figure 1:**
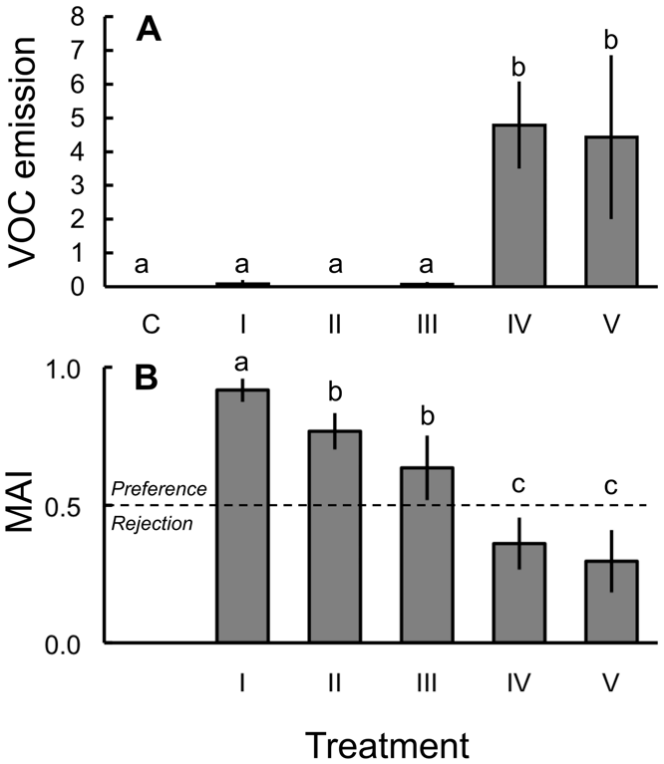
Volatile emission from plants and behavioural response of ants upon different treatments of lima bean plants (*Phaseolus lunatus*). (A) Mean total amount (±95% CI) of VOCs emitted from plants. Bars represent the total peak area relative to the peak area of an internal standard per 24 h and per 100 cm^2^ leaf surface. The following compounds have been included: (3*Z*)-hexen-1-yl acetate, (*E*,*Z*)-β-ocimene, (*R*)-(-)-linalool, DMNT, C_10_H_14_, C_10_H_16_O, indole, and TMTT. Treatments were: C) untreated control, I) LCA damage, II) scissor damage, III) pincushion damage, IV) JA-treatment, and V) LCA-herbivory and subsequent JA-treatment. Sample sizes were 8, 4, 8, 8, 13, and 4 respectively. Different letters above bars indicate significant differences (Tamhane's *post-hoc* test, P<0.05). (B) Mean acceptability index (MAI±95% CI) of *Atta colombica* workers for differentially treated test plants relative to untreated controls. Treatments like in (A). Sample sizes were 16, 15, 26, 40, and 15 respectively. Different letters indicate significant differences (Tamhane's *post hoc*-test: P<0.05).

Lima bean plants responded to the different treatments with strong and significant differences in the total amount of VOCs emitted (Fig. 1A; Welch test: F_5, 109_ = 7.864, P<0.001). VOC emission was small to negligible after a 20% reduction of their leaf area by foraging LCAs (treatment I) and mechanical damage by scissors (II) and pincushion (III) (Fig. 1A). In contrast, JA-treatment alone (IV) or combined with LCA herbivory (V) drastically increased (>20-fold) the amount of volatiles emitted.

The most dominant VOCs emitted from JA-treated plants or plants that experienced a combination of LCA damage and JA treatment were (3*Z*)-hex-3-enyl acetate, (*E*)-β-ocimene, (*R*)-(-)- linalool, (3*E*)-4,8-dimethylnona-1,3,7-triene (DMNT), (3*E*,5*E*)-2,6-dimethyl-1,3,5,7-octatetraene (C_10_H_14_), 2,6-dimethylocta-3,5,7-triene-2-ol (C_10_H_16_O), (3*E*,7*E*)-4,8,12-trimethyltrideca-1,3,7,11-tetraene (TMTT) (Table 1).

**Table 1:** Qualitative and quantitative comparison of the VOC profiles emitted from lima bean plants after different treatments.

	Relative emission of VOCs (A_VOC_ A_IS_^−1^ 100 cm^−2^ 24 h^−1^)					
Treatment	C	I	II	III	IV	V
Sample size/Compound/	n = 8	n = 4	n = 8	n = 8	n = 13	n = 4
(3*Z*)-hexen-1-yl acetate	0	0	0	0	0.81±0.74	0.17±0.14
(*E*,*Z*)-β-ocimene	0	0	0	0	2.41±0.82	3.47±3.19
(*R*)-(-)-linalool	0	0.02±0.03	0	0	0.14±0.04	0.11±0.11
DMNT^a^	0.01±0.01	0.04±0.05	0.01±0.01	0.01±0.01	0.27±0.26	0.19±0.24
C_10_H_14_ b	0	0	0	0.01±0.01	0.24±0.11	0.08±0.07
C_10_H_16_O^c^	0	0.01±0.02	0.01±0.01	0.05±0.05	0.87±0.32	0.39±0.34
Indole	0	0	0	0	0.02±0.02	0
TMTT^d^	0	0.01±0.02	0.01±0.01	0.01±0.01	0.02±0.02	0.01±0.01

a =  (3*E*)-4,8-dimethylnona-1,3,7-triene,

b =  (3*E*,5*E*)-2,6-Dimethyl-1,3,5,7-octatetraene,

c =  2,6-dimethyl-octa-3,5,7-triene-2-ol,

d =  (3*E*,7*E*)-4,8,12-trimethyltrideca-1,3,7,11-tetraene.

VOC amounts shown are mean peak areas (±95% CI) relative to the peak area of an internal standard per 24 h and per 100 cm^2^ leaf surface. Treatments were: C) untreated control, I) LCA herbivory, II) scissor damage, III) pincushion damage, IV) JA-treatment, and V) LCA-herbivory and subsequent JA-treatment.

**Figure 2:**
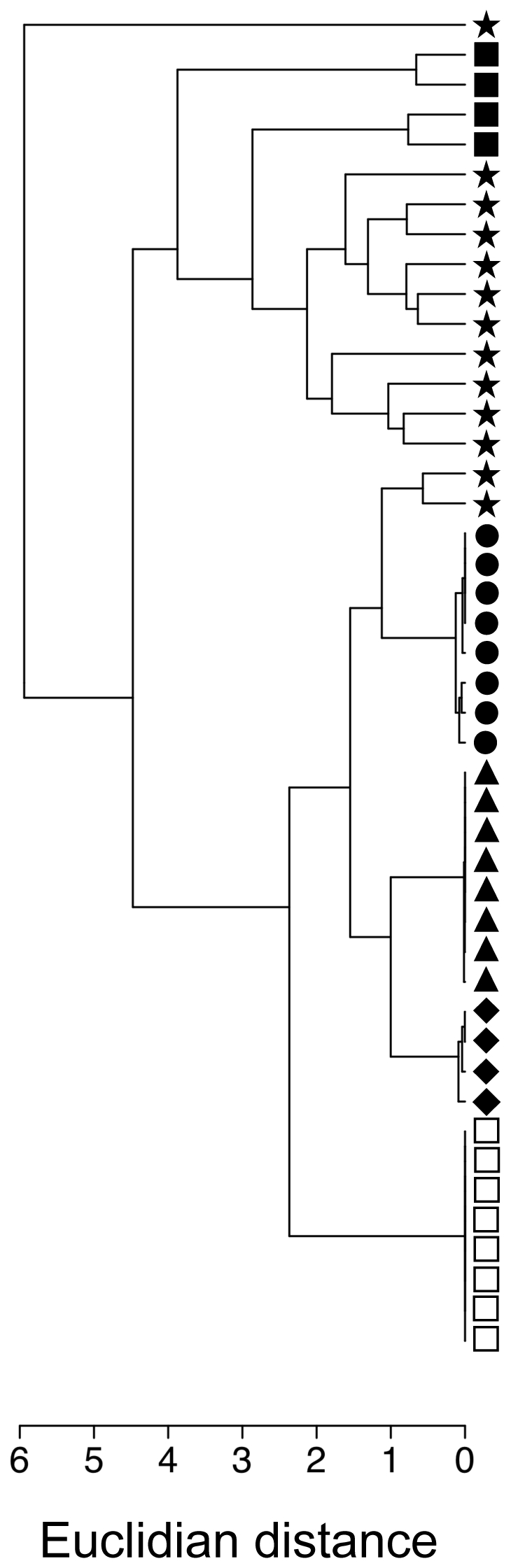
Hierarchical clustering (UPGMA) of the volatile blends emitted from differentially treated plants. Each tip corresponds to one replicate. Plant treatments were: untreated control (□); LCA herbivory (⧫); scissor damage (▴); pincushion damage (•); JA-treatment (★); LCA-herbivory with subsequent JA-treatment (▪).

According to a cluster analysis of all plants that had received one of the five treatments and of which the amounts of each of eight most dominantly emitted VOCs had been quantified (Table 1), the VOC profile emitted from LCA-damaged plants (treatment I) clustered together with undamaged controls and mechanically damaged plants (treatments II and III). In contrast, the JA-treated and ‘JA + LCA-damage’-treated plants (treatments IV and V) that showed the highest emission levels of VOCS (Fig 1A) formed a separate cluster that was distinct from all other treatments (Fig. 2).

### Food Plant Preference 24 h after LCA Damage

Determination of food plant preferences revealed a highly significant response that strongly depended on the different treatments applied (Fig. 1B; univariate ANOVA: F_4, 103_ = 10.065, P<0.001), while colony identity did not affect the model (‘ant colony’ as random factor, univariate ANOVA: F_2, 103_ = 0.315, P>0.05). LCAs clearly preferred plants they had cut 24 h before over undamaged control plants (treatment I). Mechanical damage of plants by either scissors (II) or a pincushion (III) elicited a similar, yet weaker preference over control plants (Tamhane's post-hoc test: P<0.05). Finally, foraging workers strongly discriminated against plants that either had been treated with JA alone or in combination with LCA damage (treatments IV and V) relative to control plants. Analysis of the statistical relationship between the MAIs measured and the amount of VOCs emitted revealed a highly significant negative correlation between these two parameters (Fig. 1; Spearman rank correlation: R = −0.766, n = 37, P<0.001).

### Volatile Emission 24 h after Beetle Feeding

**Figure 3:**
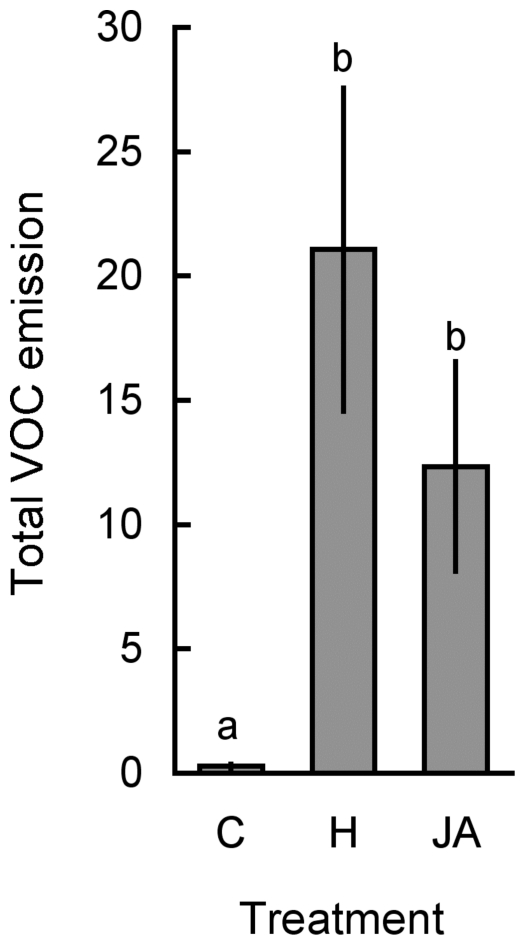
Comparison of the total amount of VOCs emitted from differentially treated lima bean plants. Plants were left undamaged (C), herbivore-damaged (H, Mexican bean beetle (*Epilachna varivestis*)), or treated with the phytohormone JA (JA). The total of the following emitted volatiles are given as mean peak area (±95% CI) relative to the peak area of an internal standard per 24 h and per gram dry weight: (3*Z*)-hexen-1-yl acetate, (*E*,*Z*)-β-ocimene, (*R*)-(-)-linalool, DMNT, C_10_H_14_, methyl salicylate, C_10_H_16_O, (*Z*)-jasmone, β-caryophyllene, TMTT. Plants of the herbivore treatment were exposed to five beetles for 24 h, which had consumed 14.2±3.5% (mean±95% CI) of the plants total leaf area (5 leaves). Sample sizes were 16, 16, and 6 respectively. Different letters indicate significant differences between treatments (Tamhane's *post-hoc* test: P<0.05).

The level of VOCs induced in the lima bean after damage of herbivores with chewing mouthparts was measured after plants have been exposed to five Mexican bean beetles for 24 h. On average, beetles had consumed 14.2±3.5% (mean±95% CI) of the plants' total leaf area during this time and there was a significant positive correlation between the leaf area damaged and the total amount of HI-VOCs emitted (Spearman rank correlation: R = 0.538, n = 18, P = 0.021). Moreover, beetle damage significantly increased the total amount of VOCs emitted after 24 h relative to control plants and to a level that was indistinguishable from JA-treated plants (Fig. 3, Welch test: F_2, 37_ = 33.152, P<0.001). Also the quantitative composition of the VOC blend emitted from beetle-damaged and JA-treated plants strongly resembled each other (Table 2). The only exception was the emission rate of β-caryophyllene from plants that have been damaged by beetles, which was ca. 10-fold increased over the emission rates of JA-treated plants (Table 2).

**Table 2:** Qualitative and quantitative comparison of the VOC profiles emitted from lima bean plants after different treatments.

	Relative emission of VOCs (A_VOC_ A_IS_^−1^ g^−1^ 24 h^−1^)		
Treatment	C	H	JA
Compound/Sample size	n = 16	n = 16	n = 6
(3*Z*)-hexen-1-yl acetate	0.03±0.02	0.5±0.2	0.4±0.3
(*E*,*Z*)-β-ocimene	0.02±0.02	2.9±1.0	2.5±1.2
(*R*)-(-)-linalool	0.05±0.04	0.9±0.3	0.3±0.2
DMNT^a^	0.08±0.04	2.5±0.9	1.9±1.0
C_10_H_14_ b	0.05±0.04	1.6±0.3	1.1±0.5
methyl salicylate	0.12±0.1	0.7±0.2	0.1±0.1
C_10_H_16_O^c^	0.09±0.07	4.3±0.9	2.1±0.9
(Z)-jasmone	0.01±0.01	0.6±0.2	1.7±0.9
β-caryophyllene	0.02±0.01	6.3±1.8	0.4±0.3
TMTT^d^	0.04±0.02	1.1±0.5	0.9±1.0

a =  (3*E*)-4,8-dimethylnona-1,3,7-triene,

b =  (3*E*,5*E*)-2,6-Dimethyl-1,3,5,7-octatetraene,

c =  2,6-dimethyl-octa-3,5,7-triene-2-ol,

d =  (3*E*,7*E*)-4,8,12-trimethyltrideca-1,3,7,11-tetraene.

VOC amounts shown are mean peak areas (±95% CI) relative to the peak area of an internal standard per 24 h and per g dry weight. Treatments were: C) untreated control, H) herbivory by five Mexican bean beetles for 24 h, and JA) JA-treatment.

### Time Course of LCA Damage over 4 d: Volatile Emission and Food Plant Preferences

**Figure 4:**
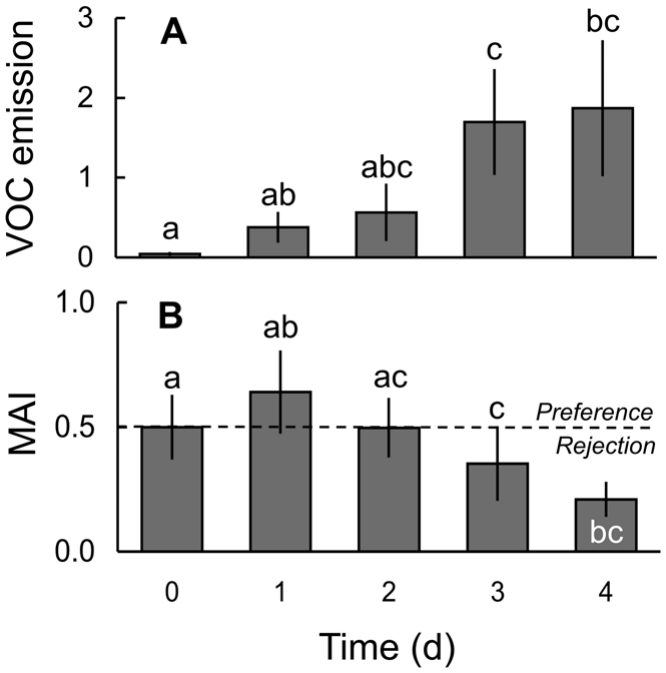
Time-course of defence induction in lima bean plants (*Phaseolus lunatus*) during 4 days of repeated LCA damage and behavioural response of ants upon exposure to LCA-damaged plants. Plants were undamaged (0 d) or ants were allowed to remove 20% of the plant's total leaf area per day for one or several days. (A) Mean total amount of VOCs (±95% CI) emitted from plants. Bars represent the total peak area relative to the peak area of an internal standard per 24 h and per 100 cm^2^ leaf surface. Compounds included are: (3*Z*)-hexen-1-yl acetate, (*E*,*Z*)-β-ocimene, (*R*)-(-)-linalool, DMNT, C_10_H_14_, C_10_H_16_O, indole, and TMTT. Samples sizes were 20, 6, 12, 12, and 7 respectively. (B) Mean acceptability index (MAI ±95% CI) of *Atta colombica* workers for differentially treated test plants (i.e. same treatments as in A) relative to untreated controls. Sample size was 11 for all comparisons. Different letters indicate significant differences (Tamhane's *post hoc*-test: P<0.05).

The amount of VOCs emitted from LCA-damaged plants 24 h post ant damage was statistically indistinguishable from undamaged controls (Fig. 4A; Welch test: F_4, 52_ = 13.172, P<0.001; Tamhane's post-hoc test: P>0.05). However, a further increase of the damage level inflicted by LCAs over the course of four days resulted in a gradual increase of the amount of VOCs emitted from LCA-damaged plants (Fig. 4A). The total amount of VOCs emitted from the so-treated plants was significantly elevated over emission levels from undamaged control plants starting at day three after the onset of the LCA damage treatment (Welch test: F_4, 52_ = 13.172, P<0.001; Tamhane's post-hoc test: P<0.05). Correspondingly, when subjected to bioassays, LCA workers distributed their foraging effort equally among two undamaged plants as well as between an undamaged plant and a plant from which LCAs had removed 20% of its leaf area 24 h before (Fig. 4B). However, offering plants that had been damaged repeatedly by LCAs over a period of three or four days prompted foraging workers to significantly discriminate against these plants relative to undamaged controls (Fig. 4B; univariate ANOVA: F_4, 35_ = 6.084, P<0.01, Tamhane's post-hoc test: P<0.05). As before, the identity of the colony did not influence the test result (‘ant colony’ as random factor, univariate ANOVA: F_3, 35_ = 0.041, P>0.05). Finally, testing the statistical relationship between the MAIs measured and the amount of VOCs emitted revealed a significant negative correlation between these two parameters (Fig. 4; Spearman rank correlation: R = −0.294, n = 46, P<0.05).

## Discussion

The aim of the present study was to verify whether the induction of anti-herbivore defences in plants affects the foraging behaviour of LCAs. To test this ‘*induced defence hypothesis*’, we used the emission of VOCs from lima bean as a model system. This plant species increases its VOC emission upon herbivore feeding [22], [34] and some of the emitted compounds have a demonstrated ant-repellent or fungicidal effect [6], [7], [35]–[37]. In fact, dual-choice bioassays indicated that foraging workers discriminated significantly against JA-treated plants, which emitted particularly high VOC levels (Fig. 1). The supposed link between the focal induced defence and the LCAs' foraging decision was further corroborated by a strong correlation between the MAIs measured and the amount of VOCs emitted. Both findings not only support our *a priori* assumption that the VOC blend emitted from induced lima bean plants should have a detrimental effect on ants and/or their mutualistic fungus, but are also in line with the ‘*induced defence hypothesis*’.

Few studies so far have investigated whether the LCAs' foraging decision is altered in response to previous plant damage and/or herbivory. In field experiments, Howard [42] detected a slight decrease in the attractiveness of experimentally scissor-cut leaves of *Spondias mombin* and *Bursera simaruba* towards *Atta colombica* workers, which, however, could not be explained with induced changes of the leaves' chemistry. Moreover, Oliveira et al. [43] observed that *Atta sexdens rubropilosa* cut significantly smaller fragments from *Eucalyptus* plants, which were previously damaged by *Thyrinteina arnobia* relative to control plants. The authors of this study interpreted this finding as a possible response to the induction of the plant's defence system. In contrast, Vasconcelos [44] did not detect a discrimination against plants that had been attacked by *Atta laevigata* in the five preceding months. Unfortunately, these studies did not provide unambiguous evidence to either support or reject the ‘*induced defence hypothesis*’.

Here we demonstrated for the first time that the experimental induction of plant defences can significantly affect the ants' foraging decision. Moreover, the emitted VOCs have been identified as one plausible mechanism that can exert this defensive effect. Even though no other inducible direct defence besides VOCs is known to contribute to the lima bean's defence syndrome [34], our experimental design does not allow to rule out the possibility that also other inducible defences may have been operating. In any case, the LCA behaviour during the dual choice bioassays (experiments 1 and 3) suggested a chemical plant-derived factor causing the observed preference/rejection response: after entering the arena used for the bioassays, a larger number of LCA foragers visited both plant individuals offered in approximately equal proportions without cutting. After a certain time, ants suddenly started to leave one plant and began to cut the respective other one. In future, it will be very interesting to further study whether VOCs are responsible for the observed repellence and if other induced plant defences can have a similar effect.

Given that LCAs were repelled from JA-induced plants, our next question was whether also their own attack could induce the plant's defence responses. Surprisingly, an individual cutting event of LCAs did not affect a plant's VOC emission rates detectably. Instead, this treatment resembled mechanically damaged rather than induced plants (Figs. 1 and 2, Table 1) both in terms of the total amount of VOCs emitted (Fig. 1A) and the qualitative and quantitative composition of the emitted blend (Table 1, Fig. 2). Consequently, the strong induction that was observed when JA treatment and LCA damage were combined, was likely due to the phytohormone treatment and not caused by the LCA damage inflicted. An analogous pattern emerged from behavioural observations in dual-choice bioassays, in which foraging workers were attracted to plants that had experienced LCA damage the previous day.

Our observation that LCA defoliation of approximately 20% did not induce significantly increased emission levels of VOCs is quite unusual and in stark contrast to previous studies. For lima bean and many other plants species such as tobacco, corn or cotton it is well-documented that herbivore damage causes a more or less pronounced increase in the emission of VOCs (for review see [45]). For example, removal of 20% leaf area by feeding *Spodoptera littoralis* larvae induced the emission of VOCs in *Zea mays* plants [46]. We could confirm this observation in control experiments with Mexican bean beetles that were allowed to feed on lima bean plants for 24 h. During this time, beetles removed on average 14% of the plants' leaf surface and induced emission levels of VOCs that were statistically indistinguishable from JA-treated plants (Fig. 3). Moreover, mechanical damage of lima bean plants by a computer-controlled device (‘*MecWorm*’) indicated that the removal of 20% of the plant's total leaf surface was sufficient to significantly increase the emission rates of many blend constituents to a level that is quantitatively comparable to the one emitted from herbivore-induced plants (i.e. feeding of *Spodoptera littoralis* and *Cepaea hortensis*) [47].

Why then did LCA damage not result in increased VOC emission rates after 24 h? The answer to this question may lay in the fact that in most previous studies herbivores with either chewing (e.g. caterpillars, beetles) or piercing (e.g. aphids, mites) mouthparts have been used. Also, the mechanical device mentioned above (i.e. ‘MecWorm’) has been programmed such that its mode of action mimics a chewing herbivore both in terms of leaf area damaged and damage time [47]. The amount of VOCs emitted 24 h after LCA damage, however, resembled the emission pattern after a singular event of mechanical damage (Figs. 1A and 2), rather than prolonged feeding of an herbivore with chewing or sucking mouthparts. Another reason for the lacking plant response could be either the absence or an insufficient contact with plant-inducing chemical factors in the ants' saliva as they are known from the regurgitate of many lepidopteran larvae [48]. Even though it is known that LCAs ingest plant juices from cut leaves [49], they may introduce less oral secretions and hence less potential VOC elicitors into the damaged leaf than e.g. Mexican bean beetles. This factor may explain why the plants' response to LCA attacks resembled mechanical damage more than induction by chewing herbivores.

Since it is known that the VOC emission after leaf damage correlates positively with the damage level inflicted ([46], [47], beetle feeding in this study), we tested whether increasing both the duration and the amount of LCA damage can induce VOC emission. Indeed, increasing LCA damage levels stepwise over the course of four days resulted in significantly increased emission rates of VOCs after three and four days relative to undamaged controls (Fig. 4A). Moreover, foraging ant workers were significantly repelled from these LCA-damaged plants (Fig. 4B) to an extent that was comparable to JA-treated plants (Fig 1B). This means that compared to e.g. chewing insect herbivores, the way LCAs damage their food plants [50] allows foraging workers to harvest larger quantities of leaves before they are recognized by the food plant. This interpretation is consistent with field observations reporting that mature leaf-cutting ant colonies focus their foraging effort on a relatively small number of plant individuals that are heavily attacked for a short period of time, until the plant is abandoned and the colony switches to use a new food plant [2], [10].

Taken together, we could demonstrate for the first time that LCA damage can induce the emission of VOCs in attacked plants. Moreover, LCA workers were strongly repelled from plants that emitted high amounts of VOCs. Hence, these observations support prior predictions made by the ‘induced defence hypothesis’. Given the taxonomically widespread distribution of inducible anti-herbivore defences in plants [20], it appears reasonable to assume that the LCA foraging activities generate a dynamic mosaic of plants at different induction stages within a colony's home range. The spatio-temporal distribution of plants at different induction levels should in turn affect the ants' foraging decisions and may thus account for several unexplained phenomena, such as the premature abandonment of still profitable leaf sources. Whether the emitted VOCs are causal for the observed ant-repellence as well as to which extend LCA defoliation can also induce other direct or indirect defence responses in the attacked plants are interesting questions that should be addressed in future studies.

Another key finding of this study is that leaf-cutting of Atta colombica workers did not induce VOC emission until three days after the first treatment, despite considerable leaf loss. This is in contrast to what is known from herbivores with chewing or piercing mouthparts, where the removal of even less leaf area is already sufficient to significantly induce VOC emission within 24 h. Hence, our results suggest that the ants' mode of leaf-cutting allows them to maximize the amount of leaf area removed before being recognized by their food plants. This finding represents a novel mechanism of how generalist herbivores thwart the recognition system of their food plants and contributes to our understanding of the polyphagy and drastic herbivorous impact of leaf-cutting ants.
